# LAMP-LFD: establishment and evaluation of a new diagnostic platform for SARS-CoV-2 rapid detection

**DOI:** 10.3389/fpubh.2026.1798338

**Published:** 2026-03-24

**Authors:** Qizhi Diao, Sha Tang, Xin Liu, Jiajia Li, Xiangmin Zhou, Yuanyu Chen, Qiongyuan Zhang, Fangyu Yang

**Affiliations:** 1 Department of Clinical Laboratory Medicine, Sanya Women and Children's Hospital Affiliated to Hainan Medical University, Sanya, China; 2 Department of Clinical Laboratory Medicine, Hainan Branch, Shanghai Children's Medical Center, School of Medicine, Shanghai Jiao Tong University, Sanya, China; 3 Department of Clinical Laboratory Medicine, The First Affiliated Hospital of Hainan Medical University, Haikou, China; 4 Department of Clinical Laboratory Medicine, University-Town Hospital of Chongqing Medical University, Chongqing, China

**Keywords:** lateral flow dipstick, loop-mediated isothermal amplification, rapid detection, SARS-CoV-2, the N gene and The ORF1ab gene

## Abstract

**Background:**

This study aimed to establish a rapid detection method for SARS-CoV-2 by integrating loop-mediated isothermal amplification (LAMP) with lateral flow dipstick (LFD) technology.

**Method:**

Specific primers targeting the nucleocapsid (N) gene and open reading frame 1ab (ORF1ab) gene of SARS-CoV-2 were designed and labeled with 6-FAM and biotin, respectively. After systematically optimizing key reaction parameters, including primer selection, primer concentration, and LAMP reaction time, the sensitivity, specificity, and clinical applicability of the method were evaluated.

**Results:**

The proposed LAMP-LFD assay enables rapid detection within 30 min with high specificity, accurately identifying the N and ORF1ab genes of SARS-CoV-2 without cross-reactivity with influenza A hemagglutinin gene, influenza B neuraminidase gene, and respiratory syncytial virus M gene. The limit of detection reached 1.892 × 10^1^ copies/μL, showing comparable sensitivity to agarose gel electrophoresis and real-time quantitative reverse transcription PCR (RT-qPCR). The results were consistent across different batches of primers and probes, demonstrating good reproducibility. When compared with RT-qPCR using 114 inactivated throat swab samples, the diagnostic agreement rate reached 95.61%.

**Discussion:**

This study provides technical support for the surveillance and control of SARS-CoV-2 infection.

## 1 Introduction

SARS-CoV-2, classified within the β-coronavirus genus, is primarily transmitted through aerosols and direct contact, exhibiting exceptional transmissibility and genetic variability that predispose to epidemic outbreaks (1, 2). Currently, SARS-CoV-2 has emerged as a predominant pathogen of community-acquired respiratory infections, alongside influenza A/B viruses and respiratory syncytial virus (RSV), frequently leading to COVID-19 pneumonia upon infection (3). The clinical presentation of SARS-CoV-2 infection closely resembles that of other common respiratory pathogens, yet requires distinct therapeutic strategies and pharmacological interventions. Consequently, rapid diagnostic identification of SARS-CoV-2 infection and timely initiation of appropriate treatment are critical for effective containment of COVID-19 epidemics.

Nucleic acid testing remains the gold standard for COVID-19 diagnosis, with real-time quantitative reverse transcription PCR (RT-qPCR) being the most widely used clinical detection method (4). Despite its high specificity and sensitivity, RT-qPCR has notable limitations, including the requirement for expensive instrumentation, stringent laboratory conditions, and well-trained personnel, as well as time-consuming and labor-intensive procedures. These constraints hinder its ability to meet point-of-care testing (POCT) demands in primary healthcare settings such as emergency departments, nursing stations, and community healthcare facilities, particularly during public health emergencies and in resource-limited environments.

In recent years, isothermal amplification technologies have demonstrated broad application prospects in the field of rapid pathogen detection due to their advantages of operational simplicity, high sensitivity, and low requirements for specialized equipment and personnel. Among these, isothermal signal amplification strategies such as Loop-mediated isothermal amplification (LAMP), Nucleic acid sequence-based amplification (NASBA), and Recombinase polymerase amplification (RPA) have achieved remarkable progress in the rapid detection of bacterial and viral pathogens, nucleic acids, and proteins (5–7). However, existing isothermal amplification methods present several limitations: NASBA requires prolonged reaction times (>90 min) (8, 9), while RPA is susceptible to aerosol contamination and typically relies on fluorescence detection equipment for result interpretation (10, 11). LAMP, utilizing the strand-displacing activity of Bst DNA polymerase with 4-6 specific primers recognizing 6–8 distinct regions of the target gene, enables exponential amplification under isothermal conditions. This technique exhibits rapid and sensitive characteristics, with some reactions completing within 10 min (12), promoting its application in SARS-CoV-2 nucleic acid detection.

Currently, two methods are commonly used for interpreting LAMP results: one relies on visual detection of white magnesium pyrophosphate precipitate formed during amplification, while the other employs SYBR Green I dye to indicate results through color change. However, both approaches have notable limitations: neither can specifically confirm LAMP amplification, as nonspecific amplification may also produce precipitate or color change; weak amplification often yields faint signals that are prone to misinterpretation; and adding dye prior to amplification may inhibit the reaction. In contrast, lateral flow dipstick (LFD) technology, with its simplicity and rapidity, has been increasingly applied in pathogen detection, especially in resource-limited settings. To address these issues, we designed six specific primers targeting conserved regions of the SARS-CoV-2 nucleocapsid (N) gene and open reading frame 1ab (ORF1ab) gene, and developed a novel diagnostic platform by innovatively integrating LAMP with LFD technology. This system enables visual result interpretation via test-line coloration and is described below.

## 2 Materials and methods

### 2.1. Clinical sample collection

Throat swab samples were collected from 48 patients with confirmed SARS-CoV-2 infection and 66 healthy controls, all inactivated with guanidine salt, in Sanya from January 2024 to December 2025. This study was approved by the Ethics Committee of Sanya Women and Children's Hospital Affiliated to Hainan Medical University.

### 2.2. Key reagents and instruments

The following gene sequences were obtained from Sangon Biotech (Shanghai, China): the N gene of SARS-CoV-2 (MN908947.3), the ORF1ab gene of SARS-CoV-2 (ON150065.2), the hemagglutinin (HA) gene of influenza A virus (FJ966982.1), the neuraminidase (NA) gene of influenza B virus (S61093.1), and the matrix (M) gene of respiratory syncytial virus (NC_001803.1, region: 3224-4180). Oligonucleotides, including primers and lateral flow strip capture probes, were also synthesized by Sangon Biotech (Shanghai, China). Bst 2.0 WarmStart polymerase was purchased from Vazyme Biotech (Nanjing, China). HAuCl_4_·3H_2_O along with streptavidin (SA) were acquired from Aladdin (Shanghai, China). Additional materials, including the nitrocellulose (NC) membrane, water absorption paper, sample pads, and binding mats, were supplied by Morsci Biotech (Guangdong, China). The fluorescent quantitative PCR kit was purchased from Daan Biotech (Guangdong, China) and the nucleic acid extraction reagent was obtained from Zhijiang Biotech (Shanghai, China).

Equipments utilized included an electric thermostatic water bath from Yiheng (Shanghai, China), a gel electrophoresis apparatus from Clinx (Shanghai, China), a clean bench from Zhicheng (Shanghai, China) and a PCR analyzer from Hongshi (Shanghai, China). Additionally, the single-point dispenser and the strip cutter were procured from Biodot (CA, USA).

### 2.3. Primer design

Based on the SARS-CoV-2 genetic sequence obtained from the National Center for Biotechnology Information database, two target regions for SARS-CoV-2 detection were identified: the N gene site and the ORF1ab gene site. Conservative regions within these sequences were determined through BLAST analysis. Following the principles of the LAMP method, primers for these conservative regions were designed using Primer Explorer V5 software (Eiken Chemical Co., Ltd. Tokyo, Japan). As shown in Tables 1, 2, three sets of primers, each comprising six primers, were designed for the N and ORF1ab genes regions, respectively. To facilitate detection, the5' end of the forward inner primer (FIP) was labeled with biotin, while the5' end of the backward inner primer (BIP) was labeled with 6-FAM. The designed primers were subsequently synthesized and labeled by a biotechnology company.

**Table 1:** Primer sets for LAMP for the LAMP-LFD detection of SARS-CoV-2 N gene.

**Primer set**	**Primer name**	**Sequence (5^′^-3^′^)**
N1	F3_N1	ACCAGGAACTAATCAGACAAG
	B3_N1	GACTTGATCTTTGAAATTTGGATCT
	FIP_N1	FAM-6TTCCGAAGAACGCTGAAGCGGAACTGATTACAAACATTGGCC
	BIP_N1	CGCATTGGCATGGAAGTCACAATTTGATGGCACCTGTGTA
	LF_N1	GGGGGCAAATTGTGCAATTTG
	LB_N1	Botin-CTTCGGGAACGTGGTTGACC
N2	F3_N2	TGGCTACTACCGAAGAGCT
	B3_N2	TGCAGCATTGTTAGCAGGAT
	FIP_N2	FAM-6TCTGGCCCAGTTCCTAGGTAGT-GACGAATTCGTGGTGGTGA
	BIP_N2	AGACGGCATCATATGGGTTGCA-GCGGGTGCCAATGTGATC
	LF_N2	CCATCTTGGACTGAGATCTTTCATT
	LB_N2	Botin-CTGAGGGAGCCTTGAATACACCAA
N3	F3_N3	CTGCACCTCATGGTCATGTT
	B3_N3	AGCTCGTCGCCTAAGTCAA
	FIP_N3	FAM-6GAGGGACAAGGACACCAAGTGTATGGTTGAGCTGGTAGCAGA
	BIP_N3	CCAGTGGCTTACCGCAAGGTTTTAGATCGGCGCCGTAAC
	LF_N3	CCGTACTGAATGCCTTCGAGT
	LB_N3	Botin-TTCGTAAGAACGGTAATAAAGGAGC

**Table 2:** Primer sets for LAMP for the LAMP-LFD detection of SARS-CoV-2 ORF1ab gene.

**Primer set**	**Primer name**	**Sequence(5^′^-3^′^)**
ORF1ab1	F3_O1	5′-TGCAACTAATAAAGCCACG-3′
	B3_O1	5′-CGTCTTTCTGTATGGTAGGATT-3′
	FIP_O1	5′-FAM6-TCTGACTTCAGTACATCAAACGAATAAATACCTGGTGTATACGTTGTC-3′
	BIP_O1	5′-GACGCGCAGGGAATGGATAATTCCACTACTTCTTCAGAGACT-3′
	LF_O1	5′-TGTTTCAACTGGTTTTGTGCTCCA-3′
	LB_O1	5′–Botin-TCTTGCCTGCGAAGATCTAAAAC-3′
ORF1ab2	F3_O2	5′-CCACTAGAGGAGCTACTGTA-3′
	B3_O2	5′-TGACAAGCTACAACACGT-3′
	FIP_O2	5′-FAM6-AGGTGAGGGTTTTCTACATCACTATATTGGAACAAGCAAATTCTATGG-3′
	BIP_O2	5′-ATGGGTTGGGATTATCCTAAATGTGTGCGAGCAAGAACAAGTG-3′
	LF_O2	5′-CAGTTTTTAACATGTTGTGCCAACC-3′
	LB_O2	5′-Botin-TAGAGCCATGCCTAACATGCT-3′
ORF1ab3	F3_O3	5′-GCTGTTGTTAAAATTTATTGTCCAG-3′
	B3_O3	5′-TATGTTAGCGCTAGCACG-3′
	FIP_O3	5′-GGTTTTCAAGCCAGATTCATTATGGCATGTCACAATTCAGAAGTAGG-3′
	BIP_O3	5′-FAM6-GTAAGGGTGGTCGCACTATTGCCCAATAGGCACACTTGTT-3′
	LF_O3	5′-CGGCAAGACTATGCTCAGGT-3′
	LB_O3	5′-Botin-CCTTTGGAGGCTGTGTGTTC-3′

### 2.4. Development of the LAMP-LFD detection system

**Figure 1:**
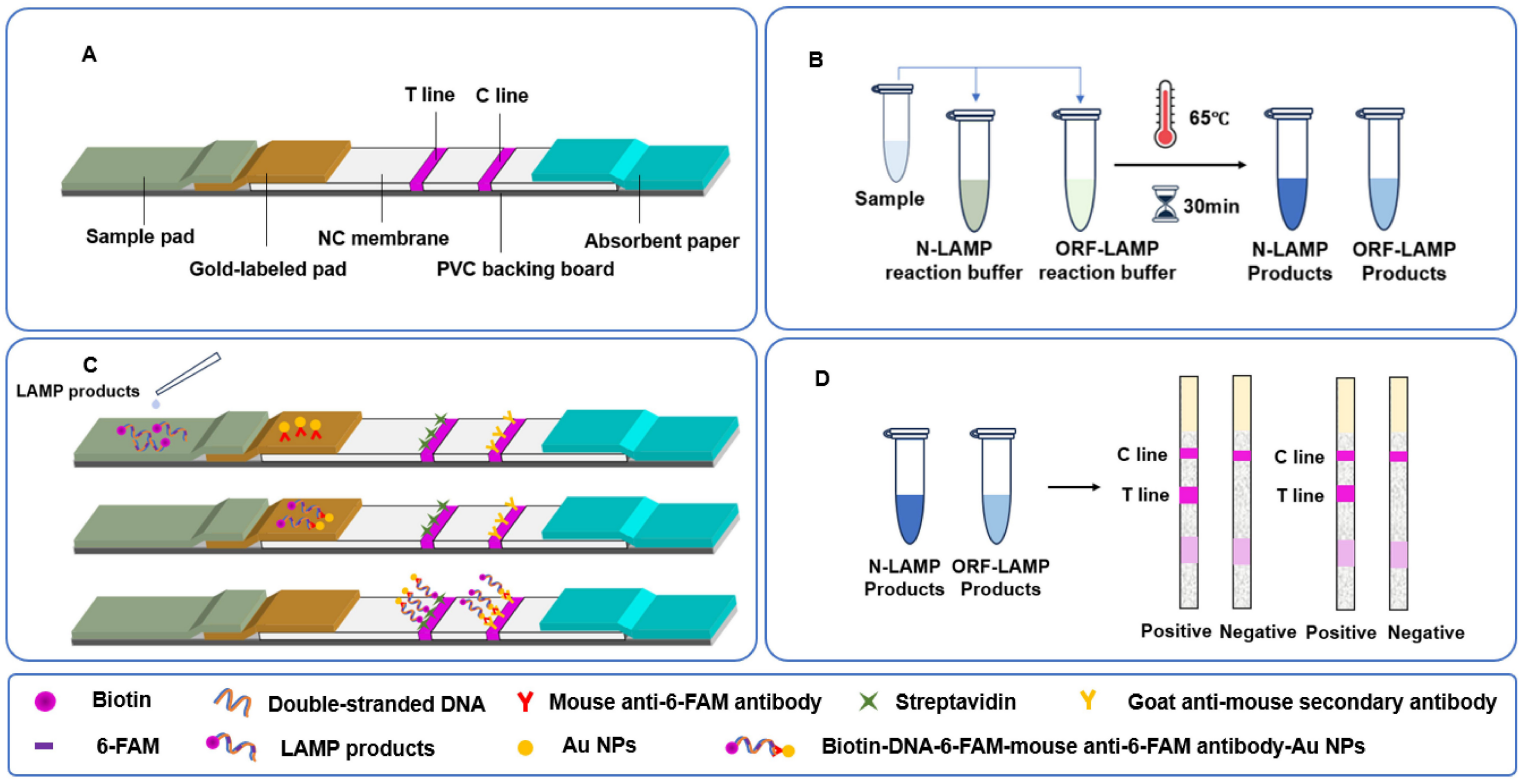
Schematic illustration of the principle of the LAMP-LFD assay design for the detection of SARS-CoV-2. **(A)** Design schematic of the lateral flow test strip. **(B)** Process of LAMP amplification. **(C)** Lateral flow immunochromatographic assay for on-site detection of the LAMP reaction product. **(D)** Interpretation scheme for lateral flow test strip results.

The composition of the LAMP amplification system is detailed in Table 3. As shown in Figure 1, the lateral flow test strip comprises several components, including absorbent paper, a NC membrane, a gold-labeled pad, a sample pad, and a PVC backing board. These components were assembled sequentially. Specifically, the NC membrane was affixed to the center of the PVC backing board, with the sample pad and absorbent paper attached to its opposite ends. The gold-labeled pad was positioned between the NC membrane and the sample pad. Following the LAMP reaction, a significant quantity of double-stranded DNA products labeled with biotin and 6-FAM was generated. These LAMP products were mixed with a loading buffer and applied to the sample pad of the test strip. Initially, the mixture specifically bound to colloidal gold nanoparticles conjugated with mouse anti-6-FAM antibodies on the gold-labeled pad, forming a complex composed of biotin-DNA-6-FAM-mouse anti-6-FAM antibody-gold nanoparticles. This complex then migrated toward the absorbent paper via capillary action, where it specifically bound to SA on the detection line. The accumulation of colloidal gold nanoparticles on the detection line produced a visible red band. Excess colloidal gold nanoparticles conjugated with mouse anti-6-FAM antibodies continued to migrate and bound to goat anti-mouse secondary antibodies on the control line, forming a second visible red band. Consequently, in the presence of the target analyte, two distinct red bands appeared on the test strip. In the absence of the target, no biotin- or 6-FAM-labeled double-stranded DNA products were generated, resulting in no color development on the detection line, while the control line remained colored. The control line serves as an internal quality control to confirm the proper functioning of the test strip.

**Table 3:** Optimal concentration of components in the LAMP amplification.

**Reaction mix**	**Concentration**	**Volume/μL**
Lyo-ready Bst DNA polymerase	40 U/μL	0.2
dNTPs	25 mM	1.4
Betaine	5 M	5
Reaction buffer	10 X	2.5
MgCl_2_	200 mM	0.75
Primer FIP	25 μM	1.6
Primer BIP	25 μM	1.6
Primer LF	10 μM	1
Primer LB	10 μM	1
Primer F3	5 μM	1
Primer B3	5 μM	1
DNA	10^8^ or 10^7^ copies/μL	1
Water,nuclease-free	–	8.15
Total volume	–	25

In this study, two separate reaction tubes were established to detect the N gene and ORF1ab gene of SARS-CoV-2, respectively. The first tube was designed to specifically amplify the N gene template without cross-reacting with the ORF1ab gene, while the second tube targeted the ORF1ab gene template exclusively, ensuring no amplification of the N gene. The LAMP reaction was conducted under optimized temperature and time conditions. Following amplification, the products were analyzed using agarose gel electrophoresis and lateral flow test strips, with the reading time for the strips strictly controlled within 10–15 min, results obtained after 15 min were considered invalid. The results from both detection methods were subsequently compared and evaluated.

### 2.5. Comparison of clinical sample detection results between LAMP-LFD and RT-qPCR

Nucleic acids were extracted from 114 clinical throat swab samples and subsequently detected using both a commercial RT-qPCR kit and the LAMP-LFD method. The agreement rate between the LAMP-LFD method and the conventional RT-qPCR method was calculated.

## 3 Results

### 3.1. Primer screening

To evaluate the synthesized primers, LAMP reactions were performed under pre-designed preliminary reaction conditions. High-concentration templates, including N gene plasmids and ORF1ab gene pseudovirus, were used for amplification. The reaction protocol consisted of a 65 °C water bath for 30 min. Sterile ddH_2_O served as the negative control. Upon completion of the amplification process, the resulting products were analyzed using agarose gel electrophoresis and lateral flow test strips. Due to the heterogeneous lengths of the LAMP amplification products, continuous ladder-like bands were observed on the agarose gel electrophoresis diagram. Primers demonstrating high specificity, robust amplification efficiency, and positive test strip results were selected for further experimental validation.

**Figure 2:**
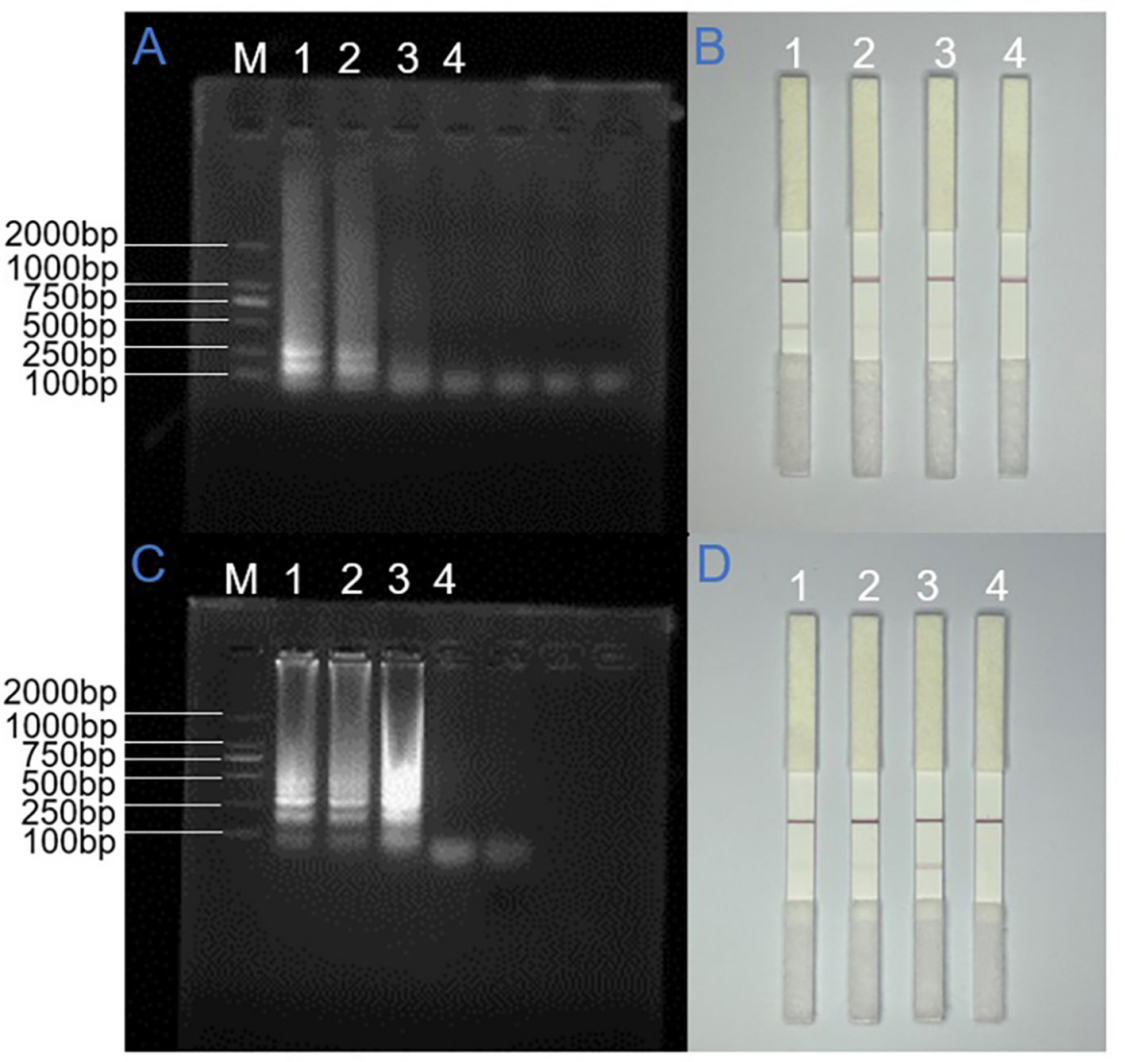
Primer screening results for SARS-CoV-2 N gene and ORF1ab gene. **(A)** and **(B)** N gene LAMP end product ran on AGE and LFD detection. **(C)** and **(D)** AGE and LFD detection of ORF1ab gene LAMP amplification product. M: DNA ladder (100–2,000 bp), 1–4: Primer 1, Primer 2, Primer 3, and no-template control.

As illustrated in Figure 2, for the SARS-CoV-2 N gene, all three primer sets produced amplification bands in the electrophoresis assay. The first primer set exhibited the most distinct ladder-like banding pattern. Correspondingly, in the colloidal gold test, the T-line for the first primer set displayed the highest signal intensity, confirming its superior binding affinity and amplification efficiency. Thus, this primer set was selected for the N gene LAMP assay.

Similarly, the electrophoretic analysis revealed that while all three primer sets generated amplification products, the third set exhibited the most distinct ladder-like banding pattern, indicating superior amplification efficiency. Corresponding lateral flow strip analysis demonstrated that the third primer set produced the most intense coloration at the T line, suggesting optimal target recognition and amplification. These results collectively indicated that the third primer set demonstrated enhanced binding affinity and amplification performance compared to the other two sets. Based on these findings, we selected the third primer set as the optimal candidate for ORF1ab gene detection in subsequent LAMP reactions.

### 3.2. Optimization of LAMP-LFD reaction conditions

#### 3.2.1. Primer concentration optimization

**Figure 3:**
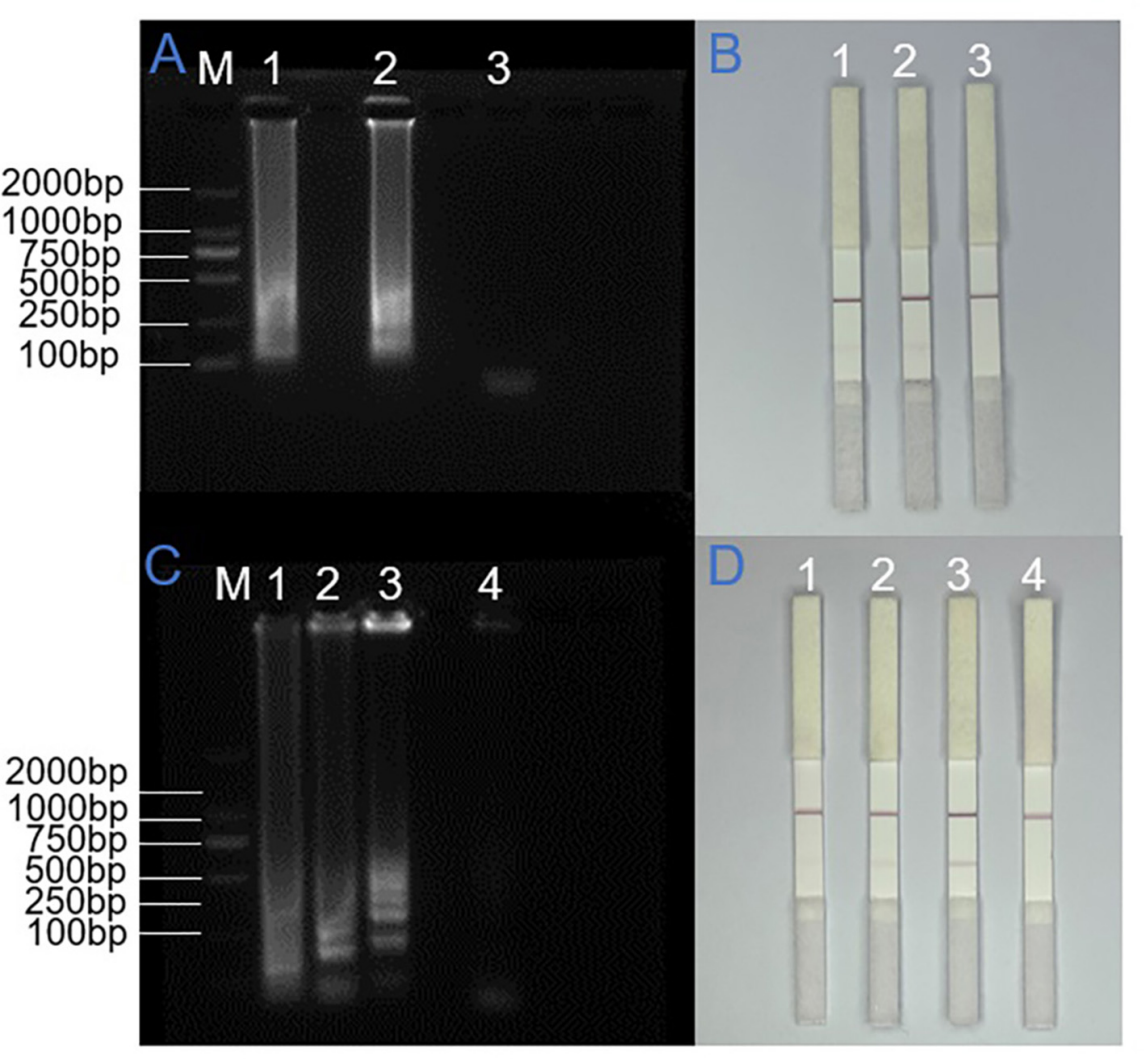
Systematic optimization of LAMP-LFD assay conditions. **(A)** and **(B)** Optimization of inner primer concentrations for N gene amplification in LAMP-AGE and LAMP-LFD detection. M: DNA ladder (100–2,000 bp), 1–3: 1.0 μM, 1.6 μM and no-template control. **(C)** and **(D)** Time-course monitoring of ORF1ab gene amplification by LAMP-AGE and LAMP-LFD. M: DNA ladder (100–2,000 bp), 1–4: 20min, 25min, 30min, and no-template control.

In the LAMP reaction, primer concentration is a critical factor influencing amplification efficiency and accuracy. Insufficient primer concentrations can reduce the reaction rate, thereby hindering amplification progress, while excessive concentrations may promote primer dimer formation. These dimers, which resemble amplification products, can lead to false-positive results and complicate the interpretation of true amplification outcomes. The fundamental mechanism of LAMP amplification highlights the critical role of inner primers in the reaction, as they initiate the formation of characteristic stem-loop DNA structures. Therefore, optimizing primer concentration is essential to ensure high reaction efficiency and reliability. To determine the optimal primer concentration for the SARS-CoV-2 N gene, amplification reactions were conducted using varying concentration ratios, with final concentrations of FIP/BIP set at either 1 μM or 1.6 μM in the standard reaction system. As shown in Figure 3, comparative analysis revealed that the higher primer concentration (1.6 μM) yielded more distinct ladder-like banding patterns in electrophoretic analysis and produced more intense coloration at the T line in lateral flow detection. Importantly, negative controls maintained specificity without any detectable false-positive signals at this concentration. Based on these findings, we established 1.6 μM as the optimal concentration for inner primers in subsequent LAMP reactions, ensuring both robust amplification and reliable specificity.

#### 3.2.2. LAMP-LFD reaction time optimization

**Figure 4:**
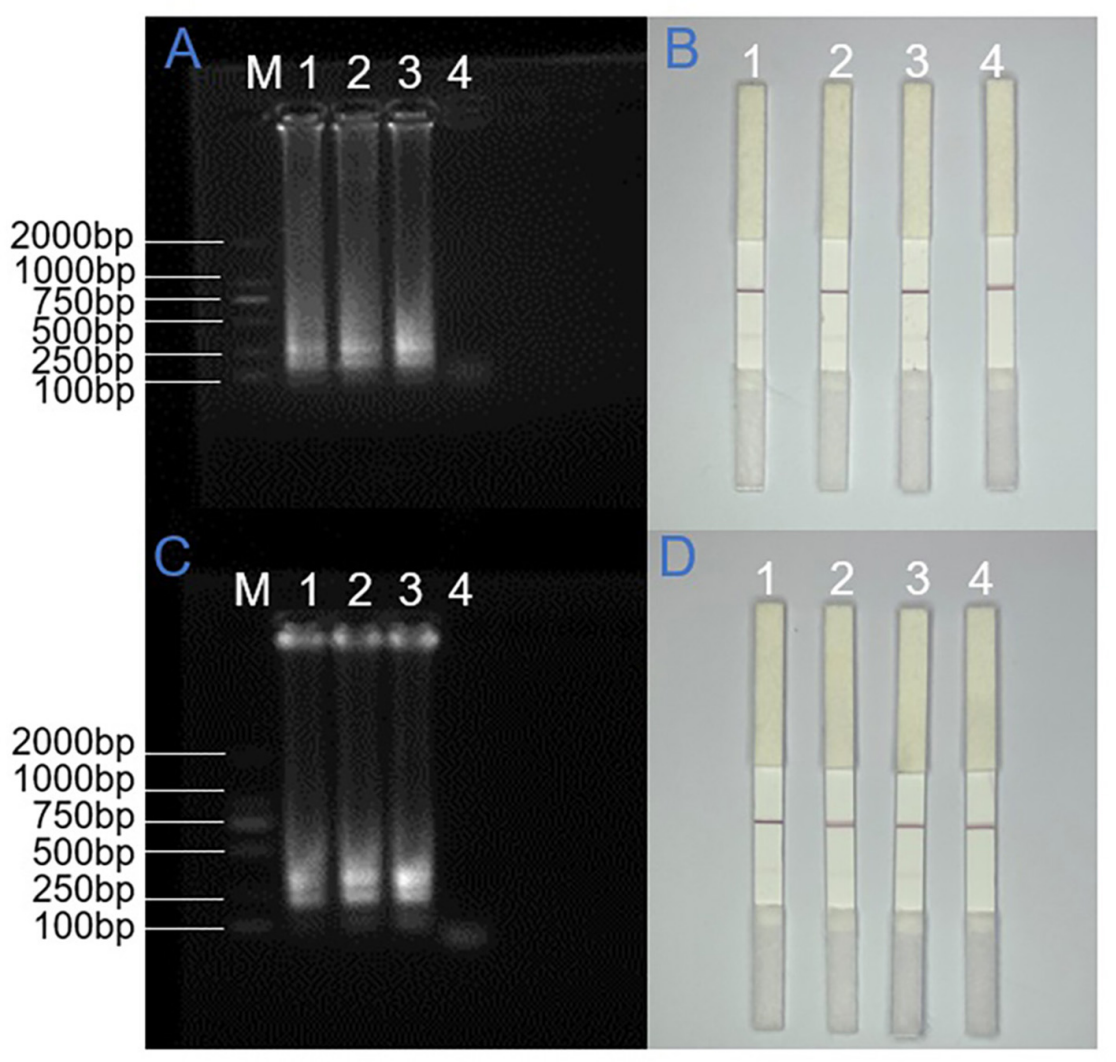
Stability validation of the LAMP-LFD detection platform. **(A)** and **(B)** Temporal reproducibility assessment of N gene amplification with AGE and LFD analysis. **(C)** and **(D)** Evaluation of temporal reproducibility in ORF1ab gene amplification using AGE and LFD detection methods.

The optimal reaction time in LAMP amplification is intrinsically linked to primer binding efficiency, with stronger primer-template interactions typically requiring shorter amplification durations. Following the determination of primer ratio and concentration, we systematically optimized the reaction time parameters. In this investigation, the SARS-CoV-2 ORF1ab gene served as the template for LAMP amplification, which was conducted at the predetermined optimal temperature of 65 °C. To establish the optimal reaction duration, we terminated the amplification process at three distinct time intervals (20, 25, and 30 min) by removing the samples from the water bath. Our experimental results, as demonstrated in Figure 4, revealed detectable amplification products after 20 min of reaction time, with complete amplification evidenced by distinct band intensity at 30 min. Corresponding lateral flow strip analysis (Figure 3) showed progressive intensification of the test line coloration with increasing reaction time, reaching maximum intensity at 30 min. This temporal analysis indicated that while initial amplification products were detectable at 20 min, optimal signal intensity and reaction completeness were achieved at 30 min. Based on these observations, we established 30 min as the optimal reaction duration for reliable and efficient LAMP amplification.

### 3.3. Reproducibility assessment of the LAMP-LFD assay

To evaluate the reproducibility of the detection system, triplicate experiments were conducted under standardized conditions using SARS-CoV-2 N gene plasmid and ORF1ab gene pseudovirus, with consistent LAMP reaction parameters and electrophoretic conditions maintained throughout. For the N gene target, three independent LAMP reaction systems were prepared at 1-h intervals using lateral flow strips from the same batch, and the amplification products were stored at 4 °C. Electrophoretic analysis and lateral flow detection were performed simultaneously after all three reactions were completed. In parallel, for the ORF1ab gene analysis, three replicate LAMP reaction systems were prepared concurrently using lateral flow strips from different batches and subjected to identical thermal cycling conditions, followed by immediate product detection. As shown in Figure 4, all replicate experiments yielded consistent results: agarose gel electrophoresis revealed distinct ladder-like banding patterns specific to both target regions, and lateral flow detection consistently produced clear red bands at the test lines. The multiple replicate experiments demonstrated 100% intra- and inter-batch concordance. All strip results were interpreted by two independent observers, indicating highly reliable result interpretation.

### 3.4. Specificity analysis of the LAMP-LFD assay

**Figure 5:**
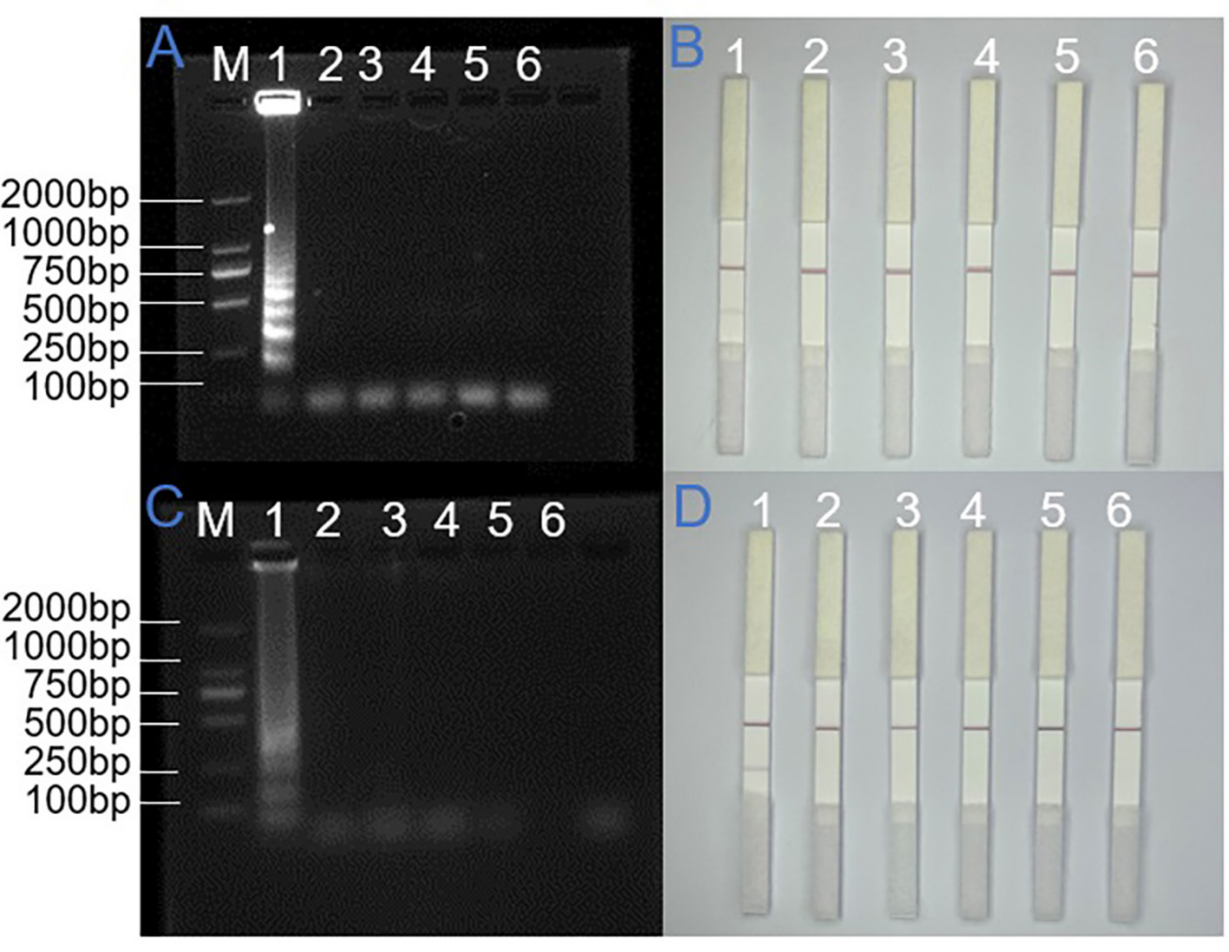
Specificity assessment of LAMP-LFD. **(A)** and **(B)** Agarose gel and Lateral flow strips showing amplification only in SARS-CoV-2 N gene sample (Number 1) with no products in non-target coronaviruses (2–4: influenza A HA gene, influenza B NA gene, RSV M gene) or SARS-CoV-2 ORF1ab gene (Number 5), Number 6: No-template control. **(C)** and **(D)** Agarose gel and Lateral flow strips confirming exclusive reactivity with SARS-CoV-2 ORF1ab gene RNA (Number 1) and negative results with other respiratory pathogens (2–5: influenza A HA gene, influenza B NA gene, RSV M gene, SARS-CoV-2 N gene; Number 6: No-template control). M: DNA ladder (100–2,000 bp).

To evaluate the specificity of the detection system, we conducted comprehensive experiments targeting both the N and ORF1ab regions of SARS-CoV-2 using the optimized reaction conditions. The specificity assessment was performed through LAMP amplification of five distinct genetic templates: the SARS-CoV-2 N gene, SARS-CoV-2 ORF1ab gene, influenza A HA gene, influenza B NA gene, and RSV M gene. As illustrated in Figure 5, for the N gene tube, the specificity evaluation results demonstrated that positive detection signals were exclusively observed when the SARS-CoV-2 N gene served as the template in the LAMP amplification system. Both agarose gel electrophoresis and LFD analyses yielded negative results for all non-target templates and negative controls. These findings confirm that the developed LAMP-based detection method exhibits high specificity for the SARS-CoV-2 N gene target, with no cross-reactivity observed against other tested genetic materials. Similarly, for the ORF1ab gene group, the specificity assessment results revealed that positive detection signals were exclusively obtained when the SARS-CoV-2 ORF1ab gene was employed as the amplification template. Both agarose gel electrophoresis and LFD analyses consistently produced negative results for all non-target templates, including SARS-CoV-2 N gene, influenza A, influenza B, and respiratory syncytial virus genetic materials, as well as negative controls. These experimental findings provide conclusive evidence that the developed LAMP-based detection platform maintains exceptional specificity for the SARS-CoV-2 ORF1ab target sequence, effectively distinguishing it from other respiratory viral pathogens.

### 3.5. Sensitivity determination of the LAMP-LFD assay

**Figure 6:**
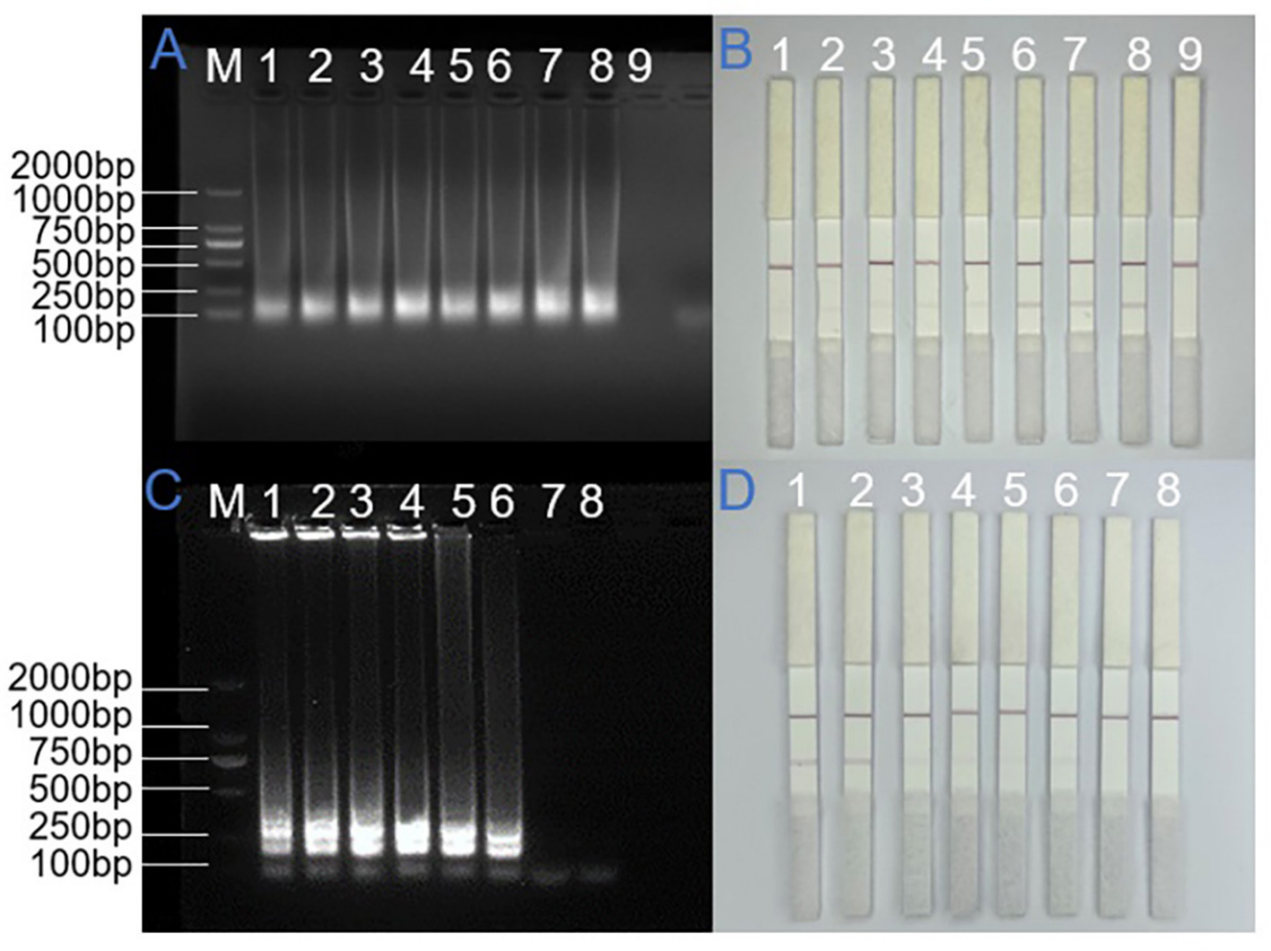
Sensitivity analysis of the LAMP-LFD assay. **(A)** and **(B)** Sensitivity evaluation using serial dilutions (10^8^ to 10^1^ copies) of N gene standard material, the corresponding LAMP products are visualized on AGE and LFD, Lane M: DNA ladder (100–2,000 bp), number 1: 10 copies, number 2: 10^2^ copies, number 3: 10^3^ copies, number 4: 10^4^ copies, number 5: 10^5^ copies, number 6: 10^6^ copies, number 7: 10^7^ copies, number 8: 10^8^ copies, number 9: non-template control. **(C)** and **(D)** Serial dilutions (10^7^ to 101 copies) of ORF1ab gene standard material were used for sensitivity validation, and the final LAMP products were assessed by AGE and LFD, Lane M: DNA ladder (100–2,000 bp), number 1: 10^7^ copies, number 2: 10^6^ copies, number 3: 10^5^ copies, number 4: 10^4^ copies, number 5: 10^3^ copies, number 6: 10^2^ copies, number 7: 10 copies, number 8: non-template control.

Ten-fold serial dilutions of SARS-CoV-2 N gene and ORF1ab gene templates were performed to establish the detection limit of the developed testing platform. As demonstrated in Figure 6, the color intensity of the LFD test line progressively diminished with decreasing viral copy numbers. For the N gene, clear electrophoretic bands and visually interpretable LFD test lines were observed at concentrations as low as 10^1^ copies/μL. For ORF1ab gene, distinct detection was achieved at 10^2^ copies/μL. These findings indicate that the LAMP-LFD method exhibits comparable sensitivity to conventional agarose gel electrophoresis analysis. Furthermore, to accurately determine the limit of detection (LOD) of the LAMP-LFD assay, 10 independent replicates were tested for each concentration of the N gene. The results showed that at a genomic RNA concentration of 10^1^ copies/μL in the reaction, 9 out of 10 replicates were positive. A probit regression analysis was performed on the results of the 10 tests using SPSS software. The calculated LOD for the LAMP-LFD reaction, at a 95% probability level, was 1.892 × 10^1^ copies/μL.

### 3.6. Application of LAMP-LFD for clinical sample detection

**Figure 7:**
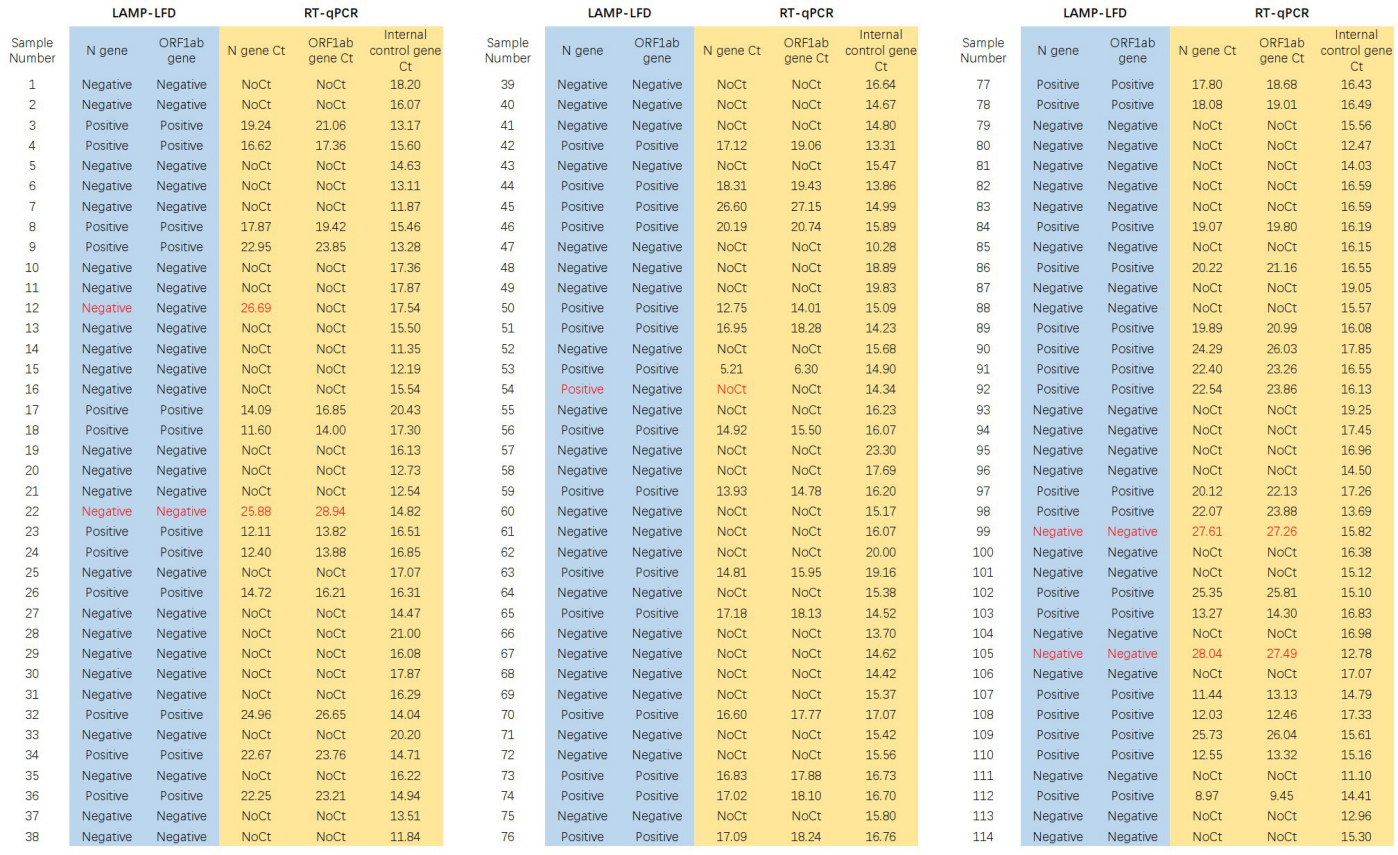
Comparison of LAMP-LFD and RT-qPCR for detection of clinical samples. Samples were classified as positive for SARS-CoV-2 when Ct values for the N gene or ORF1ab gene were ≤ 30 (with internal control Ct ≤ 30). They were classified as negative when Ct values for both N and ORF1ab genes were >30 (with internal control Ct ≤ 30).

The LAMP-LFD method and RT-qPCR were used to detect 114 inactivated pharyngeal swab samples, with RT-qPCR results serving as the gold standard. The samples included 48 confirmed SARS-CoV-2 infected patients and 66 healthy individuals. As shown in Figure 7, the LAMP-LFD method yielded positive results in 45 samples and negative results in the remaining 69 samples, which included 4 samples from confirmed SARS-CoV-2 infected individuals and 65 samples from healthy subjects. In this study, the sensitivity and specificity of the LAMP-LFD method were 91.67%, and 98.48%, respectively; the positive predictive value and negative predictive value were 97.78%, and 94.2%, respectively; and the diagnostic concordance rate reached 95.61%,the kappa statistic was 0.909, with *P* < 0.001. These results indicate that the LAMP-LFD method exhibits high concordance for SARS-CoV-2 detection, as presented in Table 4.

**Table 4:** Clinical sample validation results.

**LAMP-LFD**	**RT-qCR**		
	**Positive (**+**)**	**Negative (–)**	**Total**
Positive (+)	44	1	45
Negative **(–**)	4	65	69
Total	48	66	114

## 4 Discussion

Recent etiological surveillance data from seven regional sentinel hospitals in China reveal age-stratified pathogen distribution patterns: in pediatric populations (< 14 years), Mycoplasma pneumoniae represents the predominant pathogen (32.6% detection rate), followed by SARS-CoV-2 (18.4%); conversely, among adults (≥14 years), SARS-CoV-2 emerges as the leading pathogen (25.1% detection rate), significantly surpassing both Mycoplasma pneumoniae (14.7%) and Haemophilus influenzae (9.2%) in prevalence (13). The predominant Omicron variant in SARS-CoV-2 infection exhibits significantly enhanced transmissibility (3–5 fold higher than previous strains) due to increased spike protein–receptor binding affinity, along with substantial antigenic drift that reduces neutralizing antibody efficacy by 4–8 fold, thereby markedly boosting its immune escape capability (14, 15). To address these epidemiological challenges, we developed an innovative diagnostic platform through strategic integration of LAMP and LFD technologies. This system specifically addresses two critical limitations of conventional LAMP: implementation of pre-mixed 6-FAM labeled probes within the amplification system, achieving a significant reduction in total processing time to 30 min; and development of a closed-tube, solid-phase chromatographic detection system, which effectively mitigates contamination risks inherent in conventional open-tube operations through integrated colorimetric interpretation. Validation studies demonstrate the system's clinical utility, with visual detection sensitivity reaching 1.892 × 10^1^ copies/μL—sufficient to detect asymptomatic infections typically presenting viral loads of 10^5^-10^8^ copies/ml. Specificity testing revealed no cross-reactivity with influenza A/B viruses or RSV, while rigorous evaluation under varying experimental conditions confirmed excellent reproducibility and long-term stability.

The LAMP-LFD platform represents a significant technological advancement over conventional LAMP detection methodologies, including calcein colorimetry (16), agarose gel electrophoresis (17), magnesium pyrophosphate turbidimetry (18), and fluorescent dye-based systems (19), particularly in terms of operational efficiency and biosafety. Conventional approaches are constrained by some fundamental limitations: stringent cold-chain requirements for calcein storage, substantially increasing logistical complexity and costs; non-specific binding of fluorescent dyes (e.g., SYBR Green) to double-stranded DNA, potentially compromising amplification efficiency; time-consuming and low-throughput gel electrophoresis protocols that necessitate carcinogenic ethidium bromide (EB), posing significant biosafety concerns; substantial dependence on sophisticated instrumentation (e.g., fluorescence microscopes, electrophoresis systems), severely limiting field applicability; and inherent inability to distinguish between specific and non-specific amplification products. Our LAMP-LFD platform addresses these limitations through three strategic innovations: First, complete elimination of hazardous reagents (calcein and EB) significantly reduces environmental and operator risks. Second, implementation of a dual-line chromatographic detection system replaces conventional liquid-phase methods, enabling rapid (< 5 s) visual interpretation while maintaining enhanced sensitivity and specificity. Third, the system's minimal instrumentation requirements—utilizing only basic isothermal amplification equipmen rather than expensive quantitative PCR system or fluorescence microscopy—dramatically improve accessibility and field deployability.

Furthermore, compared to previously reported LAMP-LFD platforms, the system established in this study demonstrates distinct advantages. For instance, although the method developed by Ye et al. (20) improved sensitivity and reduced detection time, it still relied on quantitative PCR instrumentation and specialized operation. Simon et al. (21) designed primers targeting only the N gene, achieving a LOD of 100 copies/μL without clinical sample validation. The platform reported by Tang et al. (22) exhibited an LOD of 500 copies/μL. The system established by Agarwal et al. (23) showed an accuracy of only 81.66% for RNA sample detection. Moreover, the complete detection process described by Chen et al. (24) required 80 min. In contrast, our platform achieves a higher detection sensitivity, a shorter turnaround time, and a more user-friendly workflow, making it more suitable for on-site rapid screening and primary healthcare applications.

While our platform demonstrates significant diagnostic advantages, two strategic development pathways merit further exploration to enhance its clinical and epidemiological utility: Frist, expansion from single-pathogen (SARS-CoV-2) detection to multiplex pathogen identification, inspired by recent breakthroughs in multiplex LAMP technology capable of simultaneously detecting 13 HPV subtypes (25). This could be achieved through innovative development of multi-channel LFD test strips, thereby broadening the platform's diagnostic spectrum. Second, integration with smartphone-based diagnostic platforms, building upon existing systems that have established portable detection capabilities for SARS-CoV-2 and influenza A/B viruses with performance characteristics meeting CDC RT-qPCR standards (26). Considering that smartphone penetration exceeds 50% of the global population, future implementation of machine learning-based image recognition algorithms for enhanced analytical accuracy, coupled with IoT-enabled real-time data integration into public health surveillance systems, could revolutionize field-based diagnostic workflows and epidemiological monitoring.

## 5 Conclusion

In summary, the detection system we have developed establishes a rapid, accurate, and user-friendly paradigm for SARS-CoV-2 screening. It demonstrates significant public health benefits and cost-effectiveness across diverse application scenarios, particularly in resource-limited settings, primary healthcare facilities, and rapid field deployments.

## Data Availability

The original contributions presented in the study are included in the article/supplementary material, further inquiries can be directed to the corresponding authors.
